# Evidence for multisensory integration in the elicitation of prior entry by bimodal cues

**DOI:** 10.1007/s00221-012-3191-8

**Published:** 2012-07-28

**Authors:** Doug J. K. Barrett, Katrin Krumbholz

**Affiliations:** 1 School of Psychology, University of Leicester, Leicester, UK; 2 MRC Institute of Hearing Research, University Park, Nottingham, UK

**Keywords:** Exogenous attention, Intramodal, Crossmodal, Multisensory integration

## Abstract

This study reports an experiment investigating the relative effects of intramodal, crossmodal and bimodal cues on visual and auditory temporal order judgements. Pairs of visual or auditory targets, separated by varying stimulus onset asynchronies, were presented to either side of a central fixation (±45°), and participants were asked to identify the target that had occurred first. In some of the trials, one of the targets was preceded by a short, non-predictive visual, auditory or audiovisual cue stimulus. The cue and target stimuli were presented at the exact same locations in space. The point of subjective simultaneity revealed a consistent spatiotemporal bias towards targets at the cued location. For the visual targets, the intramodal cue elicited the largest, and the crossmodal cue the smallest, bias. The bias elicited by the bimodal cue fell between the intramodal and crossmodal cue biases, with significant differences between all cue types. The pattern for the auditory targets was similar apart from a scaling factor and greater variance, so the differences between the cue conditions did not reach significance. These results provide evidence for multisensory integration in exogenous attentional cueing. The magnitude of the bimodal cueing effect was equivalent to the average of the facilitation elicited by the intramodal and crossmodal cues. Under the assumption that the visual and auditory cues were equally informative, this is consistent with the notion that exogenous attention, like perception, integrates multimodal information in an optimal way.

## Introduction

Our experience of the world is derived from multiple sensory systems. The converging input provided by these systems is a powerful resource for differentiating and selecting objects for action or further analysis. However, integrating information across separate sensory systems poses the brain a computationally complex problem. For example, associating the changes in the sound of an approaching car with the expansion of its image on the retina requires the integration of binaural and retinotopic information. Prioritising the car for a behavioural response then requires the selection of this integrated information in the face of competing stimuli (e.g. other vehicles). This prioritisation is usually ascribed to selective attention, which can be “exogenously” evoked by salient perceptual events or directed towards behaviourally relevant objects in a voluntary, or “endogenous”, manner (Müller and Rabbitt 1989). The relationship between multisensory integration and attention, and the extent to which they are based on common mechanisms or rely on shared neural resources, has recently become a focus of interest in cognitive neuroscience (for reviews, see Koelewijn et al. 2010; Talsma et al. 2010). Much of the research to date is based on experiments designed to compare responses to unimodal stimuli (e.g. separately presented auditory and visual stimuli) with the response to the combined multimodal stimulus (audiovisual stimulus). Comparisons of this kind yield an index of the benefits associated with stimulation in more than one modality. Their results have provided evidence that there may be a difference in the size of these benefits for perceptual integration versus attention.

Studies of multisensory perceptual integration have shown that bimodal stimuli often evoke responses that are quantitatively different from those evoked by either of their unimodal components separately. For instance, in simple reaction time (RT) tasks, observers tend to respond to a bimodal stimulus faster than they do to either of the unimodal components alone; this has been referred to as the redundant signals effect (RSE; Forster et al. 2002; Miller 1986). The RSE is likely to be related to findings from neurophysiological and neuroimaging studies that neural activity in the superior colliculus (SC) and other brain areas is often suppressed or enhanced in response to bimodal compared to unimodal stimuli (Angelaki et al. 2009; Calvert and Thesen 2004; Gu et al. 2008; Molholm et al. 2002; Morgan et al. 2008; Stein et al. 2009; Sinnett et al. 2008; Teder-Sälejärvi et al. 2005; Werner and Noppeney 2010). The degree of bimodal enhancement or suppression has been found to depend on the temporal and spatial congruency of the unimodal stimulus components (Frassinetti et al. 2002; Stein and Stanford 2008). Typically, the size of the bimodal response cannot be predicted on the basis of the responses to either of its unimodal components (Meredith and Stein 1983; Stein et al. 2009). This suggests that bimodal perceptual integration is based upon a true combination of unimodal responses, rather than an exclusive decision based on either unimodal response alone (e.g. a “winner-takes-all” mechanism; Mulligan and Shaw 1980).

In contrast to the studies on perceptual integration, studies of multisensory attention have found little evidence to suggest that the attentional facilitation evoked by bimodal cues is different from that evoked by their unimodal components. Most of these studies measured RTs and response accuracy to cued compared to uncued targets and have found the benefits afforded by bimodal cues to be comparable to those afforded by the most effective unimodal cue alone (Santangelo et al. 2006; Spence and Driver 1999; Ward 1994). One study also measured the neural response to bimodal cues and found bimodal enhancement of the neural response in the absence of any bimodal benefit in attentional facilitation (Santangelo et al. 2008a). This suggests that the absence of benefit for bimodal cues in the previous studies was not due to a failure to induce multisensory perceptual integration. These results have been interpreted as evidence that multisensory perceptual integration and attention are based on different underlying mechanisms (Bertelson et al. 2000; Santangelo et al. 2006; Spence 2010): while multisensory perceptual integration is thought to reflect a true combination of unimodal information, multisensory attention appears more consistent with facilitation being based on a winner-takes-all competition between the unimodal cue components. This competition might take place between separate modality-specific attentional resources (Chambers et al. 2004; Duncan et al. 1997; Mondor and Amirault 1998) or between the unimodal inputs to a supramodal attention mechanism (Farah et al. 1989; McDonald et al. 2001; Zimmer and Macaluso 2007).

There is, however, at least some evidence that is inconsistent with the idea that the multisensory perceptual integration and multisensory attention are based on separate mechanisms. For instance, it has been shown that exogenous shifts of attention to cues in one modality can modulate responses to targets in another modality. This indicates that attentional resources are not exclusively unimodal (Driver and Spence 1998; McDonald et al. 2005; Störmer et al. 2009). Moreover, while bimodal cues do not elicit a larger RT benefit than their unimodal components, they have been shown to capture attention more effectively in conditions of high perceptual load (Santangelo et al. 2008b). Thus, the absence of multisensory enhancement in attentional facilitation may reflect a lack of sensitivity in the tasks and criteria used to study multisensory attention. In particular, the RT tasks used in the previous studies are determined, at least in part, by post-perceptual factors, such as criterion shifts, working memory and response preparation, some of which may be insensitive to changes in attentional facilitation as a result of multisensory integration (Meyer et al. 1988; Eskes et al. 2007). The inability to find evidence of multisensory integration in exogenous attention may also have been exacerbated by the expectation, in most studies, that multimodal cues will evoke *enhancements* in attentional facilitation. While enhanced neural responses characterise perceptual integration in some circumstances, the relationship between the neural correlates of multisensory integration (enhancement or suppression) and its behavioural consequences is not well understood (Holmes and Spence 2005; Holmes 2007). Optimal models of multisensory integration, which consider both the mean and the variability of the response, predict responses to bimodal stimuli to fall between, rather than exceed, the responses to their unimodal components. According to the maximum likelihood estimation (MLE) model, multisensory integration is based upon an average of the unimodal estimates associated with a given object, with each estimate weighted by its respective variance (Ernst and Bulthoff 2004; Ma and Pouget 2008). If multisensory attention operates on similar principles, attentional facilitation by a bimodal cue might also be expected to approximate an average of the facilitation elicited by its unimodal components.

The aim of the current study was to re-investigate the relationship between multisensory perceptual integration and multisensory attentional facilitation by comparing the facilitation elicited by bimodal and unimodal cues. In contrast to the previous studies, we used a temporal order judgement (TOJ) rather than a RT task to measure attentional facilitation. TOJs measure the perceived order of occurrence of two asynchronous target stimuli. They have been shown to be highly sensitive to manipulations of exogenous spatial attention, in that targets at cued locations are often perceived to have occurred earlier than targets at uncued locations (e.g. Shore et al. 2001; Stelmach and Herdman 1991; Zampini et al. 2005). This bias, known as “prior entry”, has been attributed to an increase in perceptual sensitivity at the cued location (Shore et al. 2001; McDonald et al. 2005). In the current study, the two target stimuli were either visual or auditory, and, in some trials, one of them was preceded by a visual, auditory or audiovisual cue. A recent study by Eskes et al. (2007) suggests that TOJs produce larger, and more reliable, cueing effects than RT tasks. TOJs might thus be expected to provide a more sensitive measure with which to investigate differences in the amount of facilitation elicited by bimodal and unimodal cues.

## Method

### Participants

A total of 22 participants (8 male, ages ranging from 20 to 43 (mean 26.6) years) took part in this study. All participants were naïve to the purpose of the study and reported normal hearing and normal, or corrected-to-normal, vision. They gave informed written consent and were paid for their participation at an hourly rate. The experimental procedures conformed to the Code of Ethics of the World Medical Association (Declaration of Helsinki) and were approved by the local ethics committee.

### Stimuli and apparatus

In order to make the auditory and visual TOJ tasks as similar as possible, we used target stimuli that differed along a categorical dimension. In addition, we required the auditory targets to be readily localisable, which meant that they had to be spectrally broad. To satisfy these constraints, we used a colour discrimination task for the visual TOJs, and a vowel discrimination task for the auditory TOJs.

The visual targets were two isoluminant (13.6 cd/m^2^) squares, one red and the other green, on a dark (1.7 cd/m^2^) background. Each square subtended 9° of visual angle. The visual stimuli were projected onto an acoustically transparent sheet, positioned at a viewing distance of 49 cm, using a floor-mounted projector (NEC WT610; London, UK). The image refresh rate was 75 Hz.

The auditory targets were the two vowels /i/ and /o/, generated using a Klatt synthesiser. Among the canonical vowels, /i/ and /o/ are the most widely separated in logarithmic formant space. The glottal pulse rates (GPRs), and thus the pitches, of two vowels differed by ±2 semitones around 100 Hz. Their first three formants were separated by ±1.25 semitones to simulate a difference in vocal tract length (VTL). These GPR and VTL differences exceed the largest differences at which the vowels would still be judged as having been uttered by the same speaker (Gaudrain et al. 2009). The auditory stimuli were digital-to-analogue converted at 44.1 kHz using an ASIO-compliant sound card (Motu 24 I/O; Cambridge, MA, USA). They were gated on and off with 10-ms cosine-squared ramps to avoid audible clicks and presented at an overall level of approximately 70 dB(A) using two Bose Cube loudspeakers (Kent, UK). The loudspeakers were mounted behind the sheet onto which the visual stimuli were projected. This set-up enabled us to present the auditory and visual stimuli from the same location.

Both the auditory and visual targets were presented at an angle of ±45° from the centre of gaze. In some conditions, one of the two targets was preceded by a visual, auditory or audiovisual cue stimulus. The visual cue was a bright (102.6 cd/m^2^) white disc that subtended 9° of visual angle. The auditory cue was a burst of Gaussian noise, presented at an overall level of approximately 75 dB(A). For the audiovisual cue, the auditory and visual cues were presented synchronously and at the same location (±45° like the targets).

Stimulus presentation was controlled using MATLAB (Mathworks, Natick, MA, USA) with the Psychophysics toolbox (Brainard 1997). The experiment was conducted in a quiet, dimly lit room.

**Table 1:** Mean JNDs in milliseconds with standard errors (in brackets) for the visual and auditory TOJs by cue condition

	Baseline	Intramodal	Crossmodal	Bimodal
Visual	46.01 (4.22)	61.59 (5.23)	39.80 (3.33)	57.60 (4.48)
Auditory	102.57 (7.72)	120.73 (9.04)	122.09 (11.23)	156.88 (22.55)

### Procedure

For both target modalities (visual, auditory), TOJs were measured in four cue conditions. In one condition (“baseline”), there was no cue. In the “intramodal” cue condition, the cue was presented in the same modality as the targets (e.g. visual cue for the visual targets), and in the “crossmodal” condition, the cue’s modality was alternate to that of the targets (e.g. visual cue for the auditory targets). In the “bimodal” condition, the targets were preceded by the audiovisual cue.

**Fig. 1:**
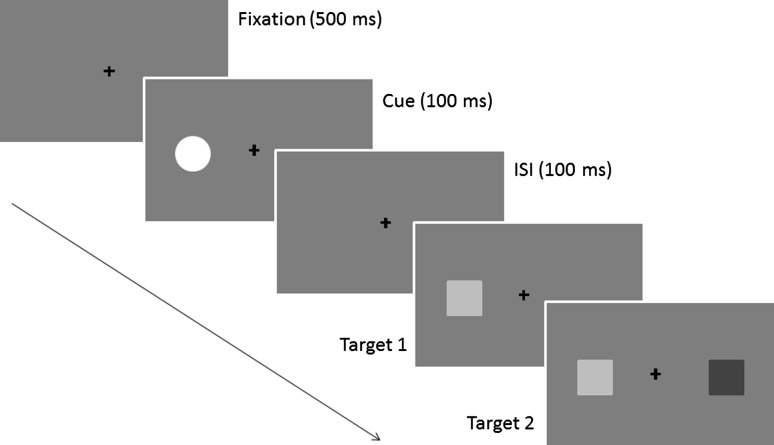
Schematic representation of one trial in the visual TOJ task. In this example, the first-appearing target is preceded by an intramodal cue. The actual visual targets were isoluminant red and green squares. The target onsets were staggered by an SOA ranging from 27 to 213 ms

Each trial began with a central fixation cross presented for 500 ms (see Fig. 1). In the cued conditions, the cue was then presented to the left or right target location for 100 ms. The first target was presented after a cue-target onset asynchrony (CTOA) of 200 ms. The CTOA was designed to simultaneously minimise both the possibility of sensory interactions between the cue and the targets [e.g. energetic masking for the auditory TOJs (Moore 2004) and “sensory facilitation” for the visual TOJs (Schneider and Bevelier 2003)] and the likelihood that participants would make saccades to the cued location prior to onset of the first target onset (Harrington and Peck 1998; Santangelo and Spence 2009). The onsets of the targets were staggered by a stimulus onset asynchrony (SOA) of 27, 53, 107, 160 or 213 ms, and the participant’s task was to identify which target had appeared first (“which-target-first” task). The first-occurring target was presented to the left or right side with equal probability. In the cued conditions, the spatial relationship between the cue and the first-occurring target was non-predictive. The targets were switched off synchronously to ensure that TOJs were based on the targets’ onsets, rather than their offsets. The duration of the longer of the two targets was always 1,000 ms. Participants were asked to judge the identity (colour or vowel identity), rather than the location of the first-occurring target, to avoid any spatial response bias (Shore et al. 2001), and their responses were recorded by the experimenter using a standard keyboard.

Previous studies have shown that orthogonal judgements are effective at eliminating first-order response bias in TOJ tasks (Spence and Parise 2010). However, concerns regarding second-order response bias have been raised by some authors (e.g. Schneider and Bevelier 2003). Two different tasks have been suggested to eliminate this second-order response bias: the simultaneity judgement (SJ) task and an alternate TOJ task, where which-target-first and which-target-second responses are averaged (Shore et al. 2001). The SJ task tends to yield much smaller prior-entry effects than the TOJ task, and there is a debate as to whether the two tasks actually measure the same underlying perceptual processes (van Eijk et al. 2008; Yates and Nicholls 2011). In contrast, the alternate TOJ task provides an effective way of eliminating second-order response bias. However, the difference between the which-target-first and which-target-second responses, which is a measure of response bias, has been shown to be small in relation to the prior-entry effect (less than 12 %; Shore et al. 2001; see Spence and Parise, for review). Furthermore, alternate tasks are likely to introduce confusion at the response-stage, as participants switch between which-target-first and which-target-second responses. In order to avoid this confusion in an already difficult task (particularly for the auditory TOJ; see “Results”), we adopted a simple which-target-first response design.

The different experimental conditions (i.e. combinations of target modality and cue condition) were run in eight separate blocks. Each block contained eight repetitions of each stimulus condition [target side (2) × SOA (5) for the baseline condition; cue side (2) × target side (2) × SOA (5) for the cued conditions]. The presentation of the stimulus conditions was randomised within each block, as was the order of presentation of blocks (i.e. experimental conditions). Participants were told to ignore the cues and asked to maintain their gaze at the central fixation throughout each trial.

### Analysis

**Fig. 2:**
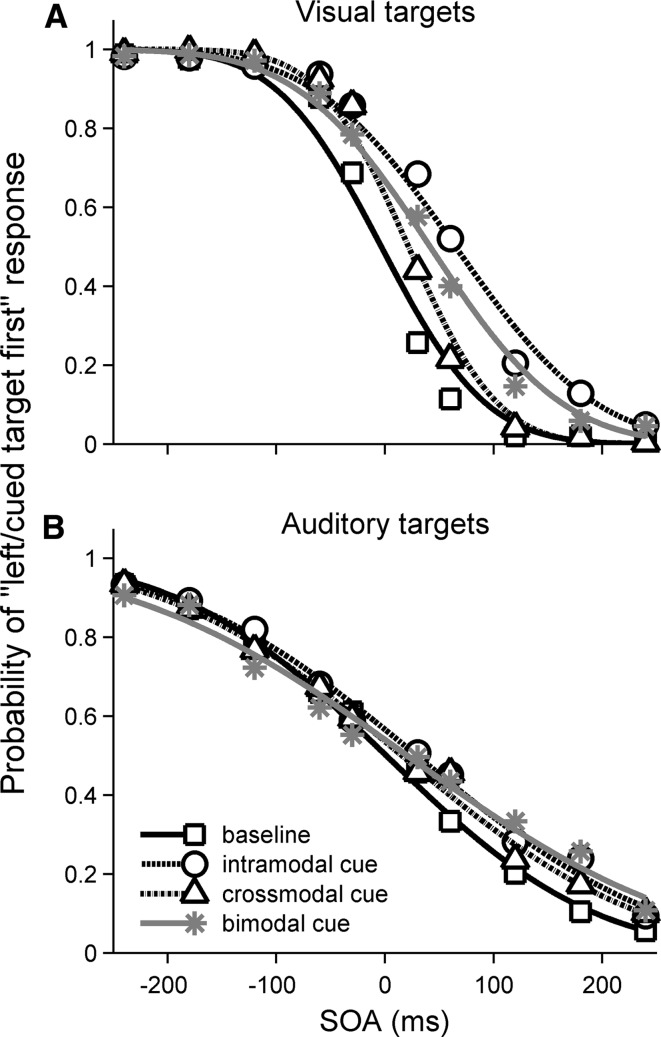
Observed data and fitted psychometric functions for the visual (**a**) and auditory (**b**) TOJ tasks. The sigmoid fitting is based upon the averaged data across participants in this illustration. The different cue conditions are represented by different symbols and line styles (see legend in **b**). For baseline trials, the ordinate shows the proportion of “left-target-first” responses, and negative SOA values on the abscissa denote targets presented first in the left visual field. For cued trials, the ordinate shows the proportion of “cued-target-first” responses, and negative SOA values denote targets presented first at the cued location, irrespective of the side of presentation

Performance in the baseline conditions was checked to ensure that, at the longest SOA (±231 ms), participants could correctly identify the first-appearing target with at least 80 % accuracy. Four participants failed to achieve this criterion and were excluded from further analysis. For the remaining participants, the results for the baseline condition were expressed in terms of the proportion of “left-target-first” responses as a function of the onset time of the left target minus that of the right (referred to as SOA in Fig. 2). For the cued conditions, the results were expressed in terms of the proportion of “cued-target-first” responses as a function of the onset time difference between the cued and the uncued target. The resulting psychometric functions were fitted with a cumulative Gaussian using the Palamedes toolbox for MATLAB (Kingdom and Prins 2010). The fitting was conducted for each participant separately. Note, however, that the fitted functions shown in Fig. 2 are based on the mean data for all participants. The goodness of fit (GoF) was estimated by bootstrapping each participant’s data 1999 times using a Monte-Carlo procedure. All participants’ responses fell well within the 95 % confidence interval around the fitted functions, indicating a good match between the fitted and measured functions. The fitted functions were then used to estimate the point of subjective simultaneity (PSS) for each participant and condition. In the baseline condition, the PSS denotes the SOA at which the left and right targets are judged to have occurred first with equal probability. The PSS for the baseline conditions would thus be expected to be close to zero. In the cued conditions, the PSS denotes the SOA at which the cued and uncued targets are judged to have occurred first with equal probability. Under the assumption that the cue facilitates target processing, the PSS for the cued conditions would be expected to be shifted towards positive SOAs (i.e. cued target occurred before uncued target). The magnitude of the shift would be expected to reflect the lead-time required for the uncued target to be perceived as having occurred simultaneously with the cued target. Next to the PSS, we also estimated the just noticeable difference (JND) in the onsets of the two targets by calculating the difference between SOAs yielding cued-target-first responses with probabilities of 0.75 and 0.5.

To compare performance across target and cue conditions, the PSS and JND estimates for each participant were entered into separate repeated-measures ANOVAs. The *p* values were Greenhouse–Geisser corrected for non-sphericity where appropriate. Post hoc comparisons were corrected for family-wise error using Holm–Bonferroni-adjusted *t* tests (two-tailed, α = 0.05).

## Results

### PSS

**Fig. 3:**
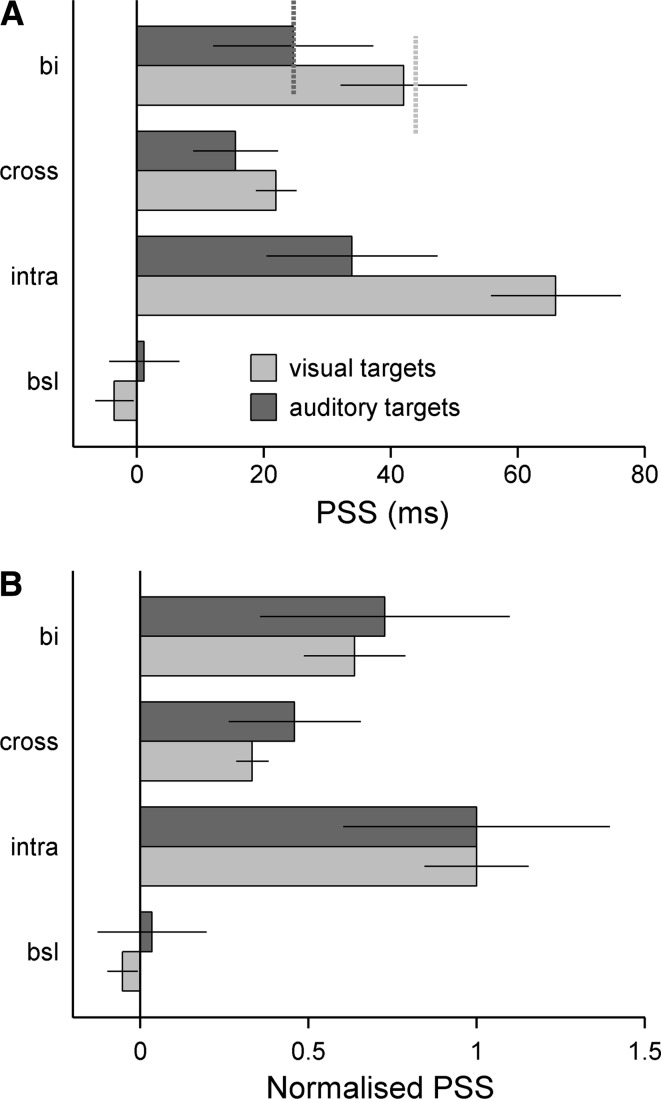
**a** Shows the average PSS for all cue conditions (*bsl* baseline, *intra* intramodal, *cross* crossmodal, *bi* bimodal). The *short dashed lines* on the set of bars showing the bimodal PSS (uppermost set) represent the mean of the intramodal and crossmodal PSS. **b** Shows the same PSS, but normalised by the PSS for the intramodal cue condition to facilitate more direct comparison between the visual and auditory TOJ tasks. *Error bars* denote the standard error of the mean

The psychometric functions for the visual and auditory TOJs were sigmoidal (Fig. 2), and the functions for the baseline conditions (no cue) were approximately mirror-symmetric about zero SOA, as expected. In contrast, the functions for the cued conditions were shifted towards positive SOAs, indicating a cue-related bias in the PSS. Figure 3a shows the mean PSS estimates derived from the individual fitted psychometric functions. It indicates that the magnitude of the bias was larger for the visual than the auditory targets (compare black and grey bars, upper panel). The bias also differed between the cue conditions, particularly for the visual targets: intramodal cues produced the largest, crossmodal cues produced the smallest, and bimodal cues produced an intermediate PSS bias. A repeated-measures ANOVA of the PSS, with factors target modality (visual, auditory) and cue condition (baseline and intermodal, crossmodal or bimodal cue), revealed a significant main effect of cue condition [*F*(3,51) = 13.64, *p* < 0.001]. The main effect of target modality was non-significant [*F*(1,17) = 2.64, *p* = 0.122], but there was a significant target modality by cue condition interaction [*F*(3,51) = 4.05, *p* < 0.012]. Post hoc tests showed that, for the visual TOJ task, all cued conditions elicited significant PSS biases compared to the baseline condition (all *p* < 0.001). Furthermore, the PSS for the intramodal cue condition was significantly larger than those for the crossmodal (*p* < 0.001) and bimodal conditions (*p* = 0.001), and the PSS for the crossmodal condition was significantly smaller than that for the bimodal condition (*p* = 0.04). For the auditory TOJ task, none of the differences between the cue conditions reached significance. This was because the cue-induced PSS biases for the auditory TOJs were considerably smaller than those for the visual TOJs, while the associated errors were larger. This difference also explains the target modality by cue condition interaction; when the PSS was normalised to the value for the intramodal cue condition in each modality (Fig. 3b), this interaction disappeared [*F*(3,51) = 0.06, *p* = 0.980]. This shows that the patterns of PSS bias across cue types for the visual and auditory target modalities were similar apart from a constant scaling factor. For both target modalities, the PSS bias elicited by the bimodal cue closely approximated the average of the biases elicited by the intramodal and crossmodal cues (visual targets: 42.06 vs. 43.99 ms, *t*(17) = 0.325, *p* = 0.749; auditory targets: 24.65 vs. 24.73, *t*(17) = 0.008, *p* = 0.99; see short dashed lines on upper-most set of bars in Fig. 3a).

### JND

Figure 2 shows that the psychometric functions for the auditory TOJs were shallower than those for the visual TOJs, indicating that the temporal order of the auditory targets was more difficult to resolve than that of the visual targets. A repeated-measures ANOVA of the mean JND estimates with factors target modality (auditory, visual) and cue condition (baseline and intramodal, crossmodal or bimodal cue) confirmed the significance of this difference (main effect of target modality: *F*(1,17) = 44.67, *p* < 0.001). There was also a significant effect of cue condition (*F*(3,51) = 5.61, *p* = 0.002). The target modality by cue condition interaction approached, but did not reach, significance (*F*(3,51) = 3.02, *p* = 0.075). Post hoc tests showed that the main effect of cue condition was driven primarily by a significantly larger JND in the bimodal compared to the baseline cue conditions (*p* = 0.022; see Table 1).

## Discussion

The aim of this study was to investigate differences in the amount of attentional facilitation associated with exogenous bimodal, intramodal and crossmodal cues for visual and auditory TOJs. For the visual TOJs, the results revealed reliable facilitation for all cue types (indexed by a spatiotemporal bias towards targets at the cued location). The visual TOJ data also revealed reliable differences in the amount of facilitation elicited by the different cue types, with the intramodal cue eliciting the largest, the crossmodal cue eliciting the smallest, and the bimodal cue eliciting intermediate facilitation. These results provide strong evidence that exogenous attentional facilitation is sensitive to the sensory information conveyed by *both* unimodal components of a bimodal cue.

In contrast to the results of the previous studies (Santangelo et al. 2006, 2008a, b; Spence and Driver 1997, 1999; Ward 1994), which have used RT tasks, the current results revealed a reliable difference in the amount of facilitation elicited by the bimodal compared to the most effective unimodal (i.e. intramodal) cue. This difference has been identified as a key criterion for multisensory integration in single-cell recordings (Stein et al. 2009). In the current study, the facilitation elicited by the bimodal cue was reduced compared to that elicited by the intramodal cue. Some of the previous RT studies have also found a tendency for a reduced bimodal cueing effect, but have not found it to be statistically reliable (e.g. Santangelo et al. 2008a). This may have been, because the difference in the amount of facilitation elicited by the intramodal and crossmodal cues was only small, and so, any reduction in the bimodal cueing effect may have been missed. In contrast, the difference was relatively large in the current study. This discrepancy between our result and that of the previous studies may, therefore, be due to the TOJ task being a more direct, and thus a more sensitive, measure of attentional modulation than RT tasks (Eskes et al. 2007). The fact that the current study used an orthogonal TOJ task means that the majority of the observed cue-induced facilitation can be attributed to attentional prioritisation or prior entry (Spence and Parise 2010). While it is possible that some proportion of the facilitation was due to second-order response bias (i.e. bias to respond to the cued target), the previous studies suggest that this effect would have been relatively small (around 10 % of the overall prior-entry effect; Shore et al. 2001). Moreover, the reduction in the facilitation elicited by the bimodal compared to the intramodal cue is inconsistent with an explanation of our data based on second-order response bias. This is because response bias would be expected to depend on the cue salience. Thus, given that the combination of the visual and auditory components of the bimodal cue would have been more, or at least equally, salient as the intramodal cue, the bimodal cue should have produced at least an equivalent response bias.

A similar argument also applies to the possibility that our results are attributable to eye movements or to sensory interactions between the intramodal cue (or cue component) and the target at the cued location. Eye movements to the cued location would not have been expected to elicit less facilitation for the bimodal than intramodal cue. Likewise, given that the bimodal cue contains the intramodal cue component, sensory interactions at the cued location would also not have been expected to elicit less facilitation for the bimodal than intramodal cue. Furthermore, in the auditory TOJ task, sensory interactions might have been expected to *reduce* the amount of facilitation elicited by the intramodal cue (through energetic masking; see Moore 2004), which is inconsistent with our finding that the intramodal cue caused the *most* facilitation. These arguments suggest that our results were not influenced by eye movements or sensory interactions. The findings of Santangelo and Spence (2009) support this interpretation. Using the same CTOA as that used in the current study, they found no evidence of any effect of eye movements or sensory interactions on cue-induced facilitation in a visual TOJ task.

In the current results, auditory TOJs were both less accurate and less susceptible to spatial cueing effects than visual TOJs. In the baseline (no cue) conditions, auditory TOJs yielded an average JND of about 103 ms compared to only 46 ms for the visual TOJs. In contrast, Kanabus et al. (2002) found comparable JNDs (of approximately 40 ms) in their auditory and visual TOJ tasks. The difference between the auditory JNDs in the current and in Kanabus et al.’s studies may be due to the tasks involving different stimulus, or feature, dimensions; the auditory targets used in Kanabus et al.’s study were tone pips presented at the same location but differing in frequency. In contrast, the auditory targets used in the current study were presented at different locations and differed in phonological (vowel) identity as well as frequency. McFarland et al. (1998) showed that JNDs for TOJs in a given modality vary depending upon the feature dimension that separates the two targets. Another important determinant of accuracy may be the extent to which the two targets temporally overlap. Kanabus et al. employed tone pips of 15-ms duration, meaning that each target was played in isolation for all but the shortest SOA. In our study, target stimuli overlapped for a variable period that depended upon the SOA on each trial. This may have made differentiating the targets more difficult.

The non-significance of the cueing effects on the auditory TOJs is also consistent with previous findings that the effect of spatial cueing on auditory RT tasks is less robust than on visual RT tasks (Barrett et al. 2010; Mondor and Amirault 1998; McDonald and Ward 1999; Spence 2010). It has been proposed that the difficulty in eliciting spatial cueing effects in hearing might be due to a fundamental difference in the way in which spatial information is represented in the auditory and visual systems. In the visual system, the mapping of non-spatial features, such as colour or orientation, is superposed onto the representation of retinotopic space. In contrast, in the auditory system, spatial and non-spatial information is processed separately from an early level onwards (Tollin 2003). This might explain why spatial information has a lesser effect on the segregation and identification of auditory compared to visual objects (Hill and Darwin 1996; Hukin and Darwin 1995). However, despite their non-significance, the PSS for the auditory TOJs revealed a similar pattern across cue types as the PSS for the visual TOJs; normalisation showed that the visual and auditory PSS only differed by a constant scaling factor and in the relative amount of variance. This indicates that the differences in the results for the visual and auditory TOJs were quantitative, rather than qualitative, and suggests auditory object recognition can be affected by spatial cueing, although to a lesser extent than visual object recognition.

The observed reduction in the amount of facilitation elicited by the bimodal compared to the intramodal cue is clearly inconsistent with a “winner-takes-all” mechanism of exogenous attention: if facilitation were determined by the most effective cue, the magnitude of the PSS bias elicited by intramodal and bimodal cues should have been equivalent (Chambers et al. 2004; Duncan et al. 1997; Mondor and Amirault 1998). The current data also argue against a strictly supramodal mechanism, which would have resulted in equivalent facilitation for intramodal and crossmodal cues (Farah et al. 1989; Koelewijn et al. 2010; Spence and Driver 1997). Instead, the amount of facilitation elicited by the bimodal cue seemed to be influenced by *both* the intramodal and crossmodal cue components. One explanation for this pattern of results is that our observers oriented to the intramodal or crossmodal cue component on half of all trials. However, this would imply that the system was switching between the more and the less effective cue component in a random fashion. Such random switching between differentially informative sources of information would be unprecedented in any other sensory or attentional functions. Thus, a more likely account of the current results is that the magnitude of the facilitation evoked by the bimodal cue was based upon a true combination of the facilitation elicited by intramodal and crossmodal cue components. This account is also more easily reconciled with evidence that attentional capture by bimodal cues is more resistant to concurrent task load (Ho et al. 2009; Santangelo and Spence 2007; Santangelo et al. 2008b) and more effective in biasing access to working memory (Botta et al. 2011). These findings, which have been attributed to an increase in the salience of bimodal compared to unimodal cues, cannot be explained by a simple switching account between exclusive, unimodal attentional resources.

The finding that the bimodal cueing effect approximated the average of the intramodal and crossmodal cueing effects suggests that multisensory combination in attentional facilitation may operate on similar principles as multisensory combination in perception. Perceptually, the combination of multimodal information has been shown to involve a weighted averaging of the multimodal stimulus components. According to the MLE model, the weights are determined by the relative precision, or inverse variance, of the representation of each component (Battaglia et al. 2003; Ernst and Banks 2002; Ernst and Bulthoff 2004; Ma and Pouget 2008). When precision differs between the unimodal components, the MLE is biased towards the most precise component. When precision is similar, the MLE reduces to a simple average of the unimodal components (Roach et al. 2006). If exogenous attention uses a similar rule to combine independent intramodal and crossmodal responses to the cue, then the magnitude of facilitation evoked by a bimodal cue would also be expected to fall between that evoked by its separate components. In the current experiment, the auditory and visual cues were both highly salient and, as cues and target always appeared at the same locations, equally informative with respect to the target locations. This suggests that the spatial information conveyed by the auditory and visual cues was similarly precise. The close approximation of the bimodal facilitation to the average of that elicited by the unimodal cues would thus seem to be consistent with an optimal model of cue combination.

The MLE model predicts that a bimodal stimulus will be represented more precisely, or reliably, than either of its unimodal components alone (Ma and Pouget 2008). This suggests that while the facilitation elicited by the bimodal cue was smaller in magnitude than that elicited by its intramodal component, its trial-to-trial reliability may have been greater. Although this cannot be determined from the current data, because the observed JNDs reflect the precision of the TOJs rather than the reliability of cueing effect, generalising the optimal averaging model of multisensory perceptual integration to multisensory attention provides a parsimonious explanation of the current results. According to this interpretation, exogenous attention is able to effectively select competing objects by combining mutually informative orienting responses across different sensory systems. As the sensitivity of different sensory systems varies with respect to the spatial and non-spatial information they encode, converging sensory information is likely to provide the most reliable means of prioritising multimodal objects for action or further analysis. This increase in the precision with which bimodal compared to unimodal cues are represented may also explain the previous findings that bimodal cues are more resistant to concurrent task load and more effective in biasing access to working memory (Botta et al. 2011; Santangelo and Spence 2007). Although further studies are required to determine whether separate unimodal orienting responses are combined in a statistically optimal way, our data suggest perception and attention may integrate multimodal information using similar rules.
